# Videolaparoscopic pancreatojejunostomy using a hybrid technique in chronic pancreatitis without significant ductal dilatation

**DOI:** 10.1590/0102-672020260000034e1963

**Published:** 2026-08-31

**Authors:** Ian Torres de LIMA, Luiz Henrique COSTA, Thiago Nogueira COSTA, Estela Regina Ramos FIGUEIRA, André Luis MONTAGNINI, Paulo HERMAN, José JUKEMURA

**Affiliations:** 1 Universidade de São Paulo, Hospital das Clínicas, Department of Gastroenterology – São Paulo (SP), Brazil.

**Keywords:** Pancreatitis, Chronic, Chronic Pain, Pancreatic Diseases, Exocrine Pancreatic Insufficiency, Pancreatite Crônica, Dor Crônica, Doenças Pancreáticas, Insuficiência Pancreática Exócrina

## Abstract

**Background:**

Chronic pancreatitis without significant main duct dilation is difficult to treat, as classic drainage procedures are less effective. Endoscopic therapy is often limited by complex anatomy, multiple strictures, stones, and the need for repeated procedures, frequently providing incomplete pain relief. Surgery offers better long-term pain control, but small-duct disease requires alternative strategies.

**Aims:**

To record the case of a 52-year-old man with alcoholic chronic pancreatitis who had progressive abdominal pain requiring opioids. Imaging showed a mildly dilated (6 mm), tortuous pancreatic duct with segmental strictures and a peripancreatic cystic lesion.

**Methods:**

Endoscopic treatment was considered suboptimal, and surgery was indicated. A hybrid laparoscopic pancreaticojejunostomy was performed, combining Izbicki’s V-shaped parenchymal excision with a longitudinal Partington-Rochelle anastomosis. The goal was to achieve effective decompression despite the small duct diameter, while preserving a minimally invasive approach.

**Results:**

Recovery was uneventful. Oral intake was resumed on the third postoperative day, and discharge occurred on the fifth. At six months, the patient had complete pain relief, a 20% reduction in insulin requirement, and improved control of exocrine insufficiency.

**Conclusions:**

This hybrid laparoscopic technique is feasible and may be an effective alternative for patients with chronic pancreatitis and small-duct disease when standard procedures are unsuitable.

## ARTICLE HIGHLIGHTS

Chronic pancreatitis with a small-calibre duct is a subgroup that is difficult to manage, in which the classic drainage techniques have limited efficacy.

We describe a hybrid videolaparoscopic technique that combines Izbicki’s V-shaped parenchymal excision with the longitudinal Partington-Rochelle anastomosis.

The approach achieved complete remission of refractory pain and withdrawal of opioids at six-month follow-up.

Pancreatic functional improvement was observed, with a 20% reduction in the insulin dose and better control of exocrine insufficiency.

## Summary

**CENTRAL MESSAGE:**

Chronic pancreatitis (CP) is a progressive and irreversible inflammation of the pancreas that results in fibrosis, loss of exocrine and endocrine function, and recurrent episodes of abdominal pain. The literature recommends that surgery be considered early in cases of intractable pain, preferably before pancreatic functional deterioration and opioid dependence. The surgical treatment of chronic pancreatitis is guided primarily by ductal anatomy and by the morphological changes of the pancreatic head. In patients with a small-calibre duct, surgical options become limited.

**PERSPECTIVES:**

Chronic pancreatitis remains a complex therapeutic challenge, especially in patients who do not present significant dilation of the main pancreatic duct. The hybrid technique described in this patient proved to be a feasible, safe, and effective alternative for controlling refractory pain, even in the context of a small-calibre duct with multiple strictures. Beyond symptomatic relief, clinical improvement was observed in the endocrine and exocrine pancreatic parameters, with sustained functional recovery at six-month follow-up. Combining classic techniques with the advances of the minimally invasive approach may broaden the available therapeutic spectrum, offering durable clinical benefits and better quality of life for patients.

## VISUAL ABSTRACT

**Figure:**
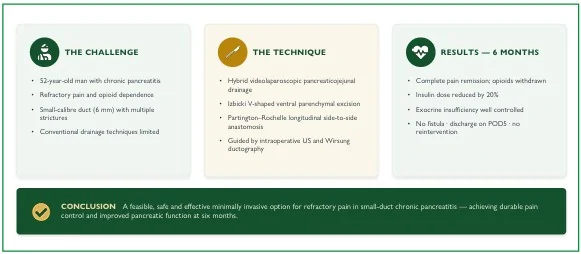

## RESUMO

**Racional:**

A pancreatite crônica sem dilatação significativa do ducto pancreático principal é de difícil manejo, pois os procedimentos clássicos de drenagem têm menor eficácia. O tratamento endoscópico é limitado por anatomia complexa, estenoses múltiplas, cálculos e necessidade de reintervenções frequentes, com alívio parcial da dor. A cirurgia oferece melhores resultados a longo prazo, mas a doença de pequeno ducto exige alternativas.

**Objetivos:**

Relatar o caso do paciente masculino de 52 anos, com pancreatite crônica alcoólica, que apresentava dor abdominal progressiva, em uso de opioides. Os exames de imagem mostraram ducto pancreático discretamente dilatado (6 mm), tortuoso, com estenoses segmentares e lesão cística peripancreática.

**Métodos:**

A via endoscópica foi considerada inadequada e indicou-se cirurgia. A técnica cirúrgica consistiu em pancreatojejunostomia laparoscópica híbrida, associando excisão parenquimatosa em “V” de Izbicki e anastomose longitudinal de Partington-Rochelle, visando descompressão eficaz mesmo em ducto de pequeno calibre.

**Resultados:**

Evolução sem intercorrências, com dieta no 3o dia e alta no 5o dia. Em seis meses, houve resolução completa da dor, redução de 20% da insulina e melhora da insuficiência exócrina.

**Conclusões:**

A técnica híbrida laparoscópica é factível e pode ser alternativa eficaz na pancreatite crônica com pequeno ducto.

## INTRODUCTION

 Chronic pancreatitis (CP) is a progressive and irreversible inflammation of the pancreas that results in fibrosis, loss of exocrine and endocrine function, and recurrent episodes of abdominal pain^7^. The main causes include chronic alcohol consumption, hereditary pancreatitis, ductal obstruction, and autoimmune, metabolic, and idiopathic causes. Its pathogenesis involves a cycle of inflammation, ductal obstruction, intraductal hypertension, and neuronal injury, which contributes to refractory chronic pain^5^. 

 Diagnosis requires clinical evaluation, laboratory tests, and imaging. Surgical indications include refractory abdominal pain, duodenal or biliary obstruction, pseudocysts, and suspected malignancy. The literature recommends that surgery be considered early in cases of intractable pain, preferably before pancreatic functional deterioration and opioid dependence^7,9^. The ideal timing is controversial, but there is growing evidence that early surgery may result in better pain control and preservation of pancreatic function. Such an approach allows preservation of the central mechanisms of nociceptive modulation, preventing chronification of the painful stimulus and the development of central sensitisation — a phenomenon that renders pain refractory and significantly hampers symptom management in the advanced stages of the disease^2,8^. 

 The surgical treatment of chronic pancreatitis is guided primarily by ductal anatomy and by the morphological changes of the pancreatic head^9^. Longitudinal pancreaticojejunostomy (the Partington-Rochelle technique) is indicated in patients with uniform dilation of the main pancreatic duct (≥7 mm) and no inflammatory mass. The Frey and Beger techniques, which combine limited resection of the pancreatic head with ductal drainage, are preferred in cases with ductal dilation associated with inflammatory hypertrophy of the pancreatic head. Pancreaticoduodenectomy (the Whipple procedure) or distal pancreatectomy is indicated in segmental disease or when malignancy is suspected. In patients with small-duct disease or diffuse pain refractory to previous treatments, total pancreatectomy with islet autotransplantation (TPIAT) may be considered^1,3,4,10^. In patients with a small-calibre duct, the surgical options become limited. 

 Endoscopic treatment, although minimally invasive, has limitations in cases of multiple strictures, impacted stones, or unfavourable anatomy^2,4^. Studies show limited success with balloon dilation, lithotripsy, and stenting in patients with multiple strictures and refractory pain. Moreover, the complication rates are relevant, including acute pancreatitis, perforation, and bleeding. Symptom recurrence and the need for multiple interventions also reduce its long-term efficacy. Surgery is associated with a higher proportion of patients achieving complete or partial pain relief (75–85% for surgery vs. 32–39% for endoscopy), with statistically significant differences and a moderate level of evidence^2,7,11^. In addition, surgery results in fewer subsequent procedures and better quality of life, without an increase in complications, mortality, or a negative impact on exocrine and endocrine pancreatic function^10^. 

 Surgery has a well-established role in controlling the symptoms of chronic pancreatitis, particularly in pain relief, and may also have an impact on preserving and even partially improving pancreatic function. Although complete reversal of exocrine and endocrine insufficiency is uncommon, approximately 10–20% of patients may show improvement in endocrine function, and 20–30% report improvement in symptoms related to exocrine insufficiency^11^. Adequate surgical treatment may halt the progression of pancreatic degeneration, reduce persistent inflammation, and improve quality of life^2,7,11^. 

 We propose an innovative videolaparoscopic pancreaticojejunal drainage technique that combines elements of the Puestow/Partington-Rochelle technique with that described by Izbicki et al.^5^ This approach aims to benefit patients with chronic pancreatitis, refractory pain, and a duct that is not markedly dilated but presents multiple strictures along its course. The Izbicki et al. technique, with longitudinal Vshaped excision of the ventral parenchyma, enables decompression even in narrow main ducts, also favouring drainage of the secondary ducts in the excised region. Combining this with lateralisation of the jejunal drainage aims to widen the area of ductal decompression, favouring pancreatic flow^5^. The laparoscopic approach adds advantages such as less postoperative pain and faster recovery^6,9^. The study was approved by the Ethics Committee of the Institution (CAAE number IRB01232/2025). 

### Surgical technique

 We present the case of a 52-year-old male patient with a history of chronic alcohol use (approximately 60 g of ethanol/day for 13 years, abstinent for eight years), active smoking, and diabetes mellitus managed with insulin therapy. 

**Figure 1:**
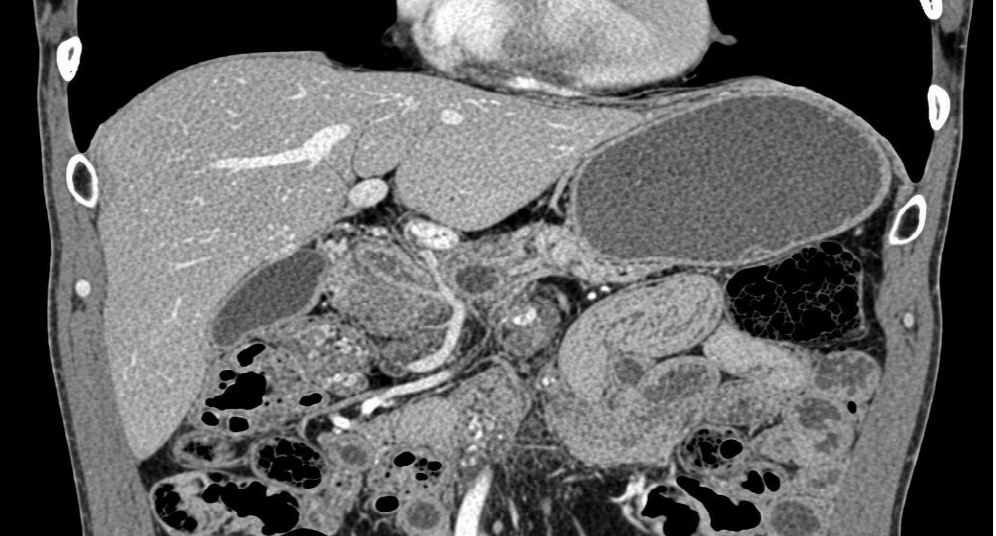
Computed tomography showing findings consistent with chronic pancreatitis. Source: Personal archive.

**Figure 2:**
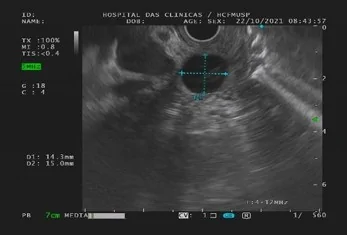
Endoscopic ultrasound confirming the presence of chronic pancreatitis. Source: Personal archive.

 The patient also had exocrine pancreatic insufficiency under regular use of pancreatin — 50,000 IU three times daily. In the two months preceding the surgical approach, he developed worsening abdominal pain, requiring escalation of analgesia with the introduction of opioids. He had had no hospital admissions in the previous two years. Laboratory investigation showed biochemical parameters within normal limits, including adequate glycaemic control (normal glycated haemoglobin). Abdominal computed tomography revealed findings consistent with chronic pancreatitis: a main pancreatic duct with irregular contours, areas of segmental stenosis with mild dilation, as well as atrophy and heterogeneous enhancement of the pancreatic tail (Figure 1). Endoscopic ultrasound confirmed the presence of chronic pancreatitis according to the Rosemont criteria, identifying a dilated main pancreatic duct (up to 6 mm) in the neck, body, and tail, in addition to a rounded, anechoic, encapsulated cystic image without septations, measuring 15 × 14 mm, located in the peripancreatic region adjacent to the pancreatic neck (Figure 2). Given the refractory pain, the need for opioids, and anatomical findings consistent with ductal strictures and tortuosity, surgical intervention was indicated after multidisciplinary discussion and patient consent. 

 The pancreaticojejunal drainage was performed videolaparoscopically, combining elements of the Partington-Rochelle and Izbicki techniques^1,5^. After release of the adhesions and adequate pancreatic exposure, the duct of Wirsung was identified using intraoperative ultrasonography and Wirsung ductography. The longitudinal opening of the duct extended from the region of cystic dilation to the pancreatic tail. In the areas of more significant stenosis, ventral parenchymal excision was performed as described in the Izbicki technique — the stenotic area was repaired with 2–0 polypropylene suture using an X-stitch, followed by section of the area with bipolar scissors. A hand-sewn side-to-side pancreaticojejunal anastomosis was then fashioned in a single layer with absorbable suture, together with an entero-anastomosis 30 cm from the pancreatic drainage. 

 Pitfalls of the technique include the difficulty in identifying the pancreatic duct in patients with minimally dilated ducts — in cases of a thin pancreatic duct, the combined use of intraoperative ultrasonography and Wirsung ductography is a valuable resource for precisely defining the point of opening of the main duct during surgery, reducing the risk of inadequate exposure. There is a risk of bleeding during dissection of the pancreatic parenchyma, in addition to the technical challenge of fashioning a safe anastomosis in a tortuous duct of variable calibre. Resection of the stenosed area also requires precision to avoid damage to the remaining parenchyma. 

## RESULTS

 The patient had an excellent immediate postoperative course. He remained fasting for the first 24 hours, with antibiotic prophylaxis using cefazolin maintained for 48 hours according to the institutional protocol. The insulin dose had to be reduced by 60% owing to fasting. On the first postoperative day, the abdominal drain output was clear serosanguineous fluid, with a volume of 15 mL/24 h. A creamy oral diet was started, with good acceptance. On the second postoperative day, the diet was adjusted to light and, on the third day, to soft, without complications. The patient was discharged on the fifth postoperative day, still with the abdominal drain, with no evidence of pancreatic fistula and with serum and abdominal-fluid amylase values within normal limits. 

 At six-month outpatient follow-up, the patient is in excellent clinical condition, with complete resolution of pain and no need for analgesics. He has good glycaemic control, with maintenance of insulin therapy but a 20% reduction in the usual dose. Symptoms related to exocrine pancreatic insufficiency remain well controlled with continuous use of pancreatin, albeit at a lower dose (25,000 IU three times daily). There was no evidence of surgical complications or need for reintervention. 

## DISCUSSION

 Chronic pancreatitis is a complex condition in which refractory pain is frequently associated with ductal obstruction, intrapancreatic hypertension, and perineural inflammation^10^. Consistent evidence demonstrates that the surgical approach, especially when performed early, offers superior results to endoscopic treatment in terms of durable pain relief, reduced opioid use, and a lower reintervention rate^5,9^. 

 The traditional surgical options are largely guided by the calibre of the main pancreatic duct^7^. The Puestow technique, later modified by Partington and Rochelle, is widely accepted as the standard surgical treatment for patients with a dilated duct (>7 mm), with pain-control success rates ranging between 70 and 85%^2,5^. However, approximately 30% of patients with chronic pancreatitis do not present significant ductal dilation, which limits the application of conventional techniques and challenges surgeons to seek safe and effective alternatives^2^. 

 The technique proposed by Izbicki, described in 1998, emerged as a response to these complex cases^1,5^. It consists of longitudinal V-shaped excision of the ventral pancreatic parenchyma, which allows broad exposure of the secondary ducts, promoting effective decompression even in the absence of significant ductal dilation. Subsequent studies demonstrated that this approach provides pain relief in up to 85% of patients, with results comparable to those obtained with the Partington-Rochelle technique in dilated ducts^1,4^. 

 Endoscopic treatment, although appealing for being minimally invasive, has important limitations. Technical success rates for stent placement or dilation of multiple strictures are below 60% in clinical series, and complications such as perforation, bleeding, and acute pancreatitis are described in up to 15% of cases^10,11^. Moreover, the long-term results reveal a greater need for reinterventions and a lesser impact on reducing opioid use compared with surgery^5,9,10^. 

 The hybrid technique presented in this report combines the principles of the Izbicki and Partington-Rochelle techniques, adapted to the laparoscopic approach. The rationale is to expand the decompression area of the main duct and the secondary ducts through V-shaped parenchymal excision combined with a longitudinal side-to-side jejunal anastomosis, promoting continuous and efficient pancreatic flow. This technique may significantly reduce episodes of acute exacerbation related to focal pancreatitis in the secondary ducts by promoting broader and more effective drainage of these structures^1^. The laparoscopic approach confers additional benefits, such as a shorter hospital stay, less postoperative pain, and early functional recovery^6,9^. 

 The choice of this technique was based on the imaging findings: a duct with limited dilation (6 mm), marked tortuosity, and segmental strictures, in addition to refractory painful symptoms. Surgery resulted in complete remission of pain at six-month follow-up, with a 20% reduction in insulin requirement and adequate control of exocrine pancreatic insufficiency. These outcomes reinforce the role of surgery not only in pain control, but also in stabilising pancreatic function. 

 In summary, this report contributes a feasible and safe technical proposal for a subgroup of patients with chronic pancreatitis whose management is traditionally challenging. Prospective multicentre studies are needed to validate this approach and to compare its outcomes with the established techniques. 

## CONCLUSIONS

 Chronic pancreatitis remains a complex therapeutic challenge, especially in patients who do not present significant dilation of the main pancreatic duct. The hybrid technique described in this patient — combining elements of the Izbicki et al.^5^ and Partington-Rochelle techniques via the videolaparoscopic route — proved to be a feasible, safe, and effective alternative for controlling refractory pain, even in the context of a small-calibre duct with multiple strictures^1,4,5^. Beyond symptomatic relief, we observed clinical improvement in the endocrine and exocrine pancreatic parameters, with sustained functional recovery at six-month follow-up. 

 This report reinforces the importance of individualised surgical strategies adapted to each patient’s ductal anatomy. Combining classic techniques with the advances of the minimally invasive approach may broaden the available therapeutic spectrum, offering durable clinical benefits and better quality of life for patients. Future clinical trials will be essential to consolidate the role of this technique in the therapeutic algorithm of chronic pancreatitis. 

## Data Availability

The datasets generated and/or analyzed during the current study are available from the corresponding author upon reasonable request.
